# Supplementary material for: BI 1015550 Improves Silica-Induced Silicosis and LPS-Induced Acute Lung Injury in Mice
Source: Molecules. 2025 Mar 14;30(6):1311. doi: 10.3390/molecules30061311 (PMC11946787; doi:10.3390/molecules30061311)
Supplement: Supplementary file 1 [file molecules-30-01311-s001.zip › molecules-3486906-supplementary.pdf]

Supplemantal information

Synthesis of BI 1015550

BI 1015550 was prepared with reference to the method of Pouzet P. et al.[30] First, amine 2 hydrochloride was used as the starting material, and the intermediate 3 was obtained by replacing chloride at the 4-position of compound 1. Then, the sulfide in compound 3 was enantioselectively oxidized to obtain chiral sulfoxide 4. Subsequently, through the SNAr reaction, the chloride at the 2-position of the pyrimidine in compound 4 was replaced by secondary amine 5 to obtain the target compound BI 1015550 (Scheme S1).

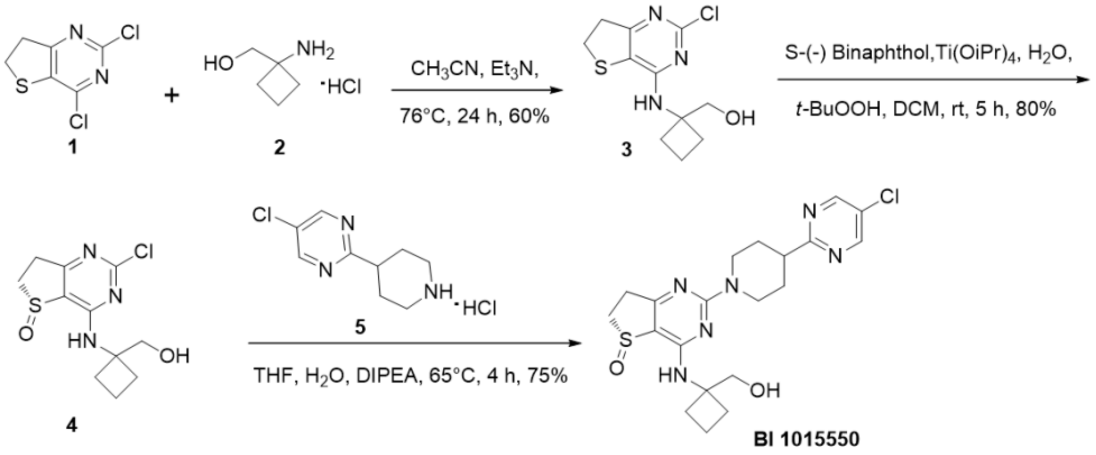

Scheme S1. The Synthesis of BI 1015550.

To a solution of compound **1** (4.14 g, 20 mmol) in acetonitrile (40 mL) were added compound **2** (3.03 g, 22 mmol) and triethylamine (8.4 mL, 60 mmol), the resulting mixture was heated to 76 °C and stirred for 12 h. Then the reaction mixture was cooled to room temperature, diluted with H_2O (50 mL) and ethyl acetate (150 mL). The layers were separated, and the aqueous layer was extracted with ethyl acetate (2×60 mL). The combined organic layers were washed with brine (2×30 mL), dried over Na_2SO_4, filtered and concentrated. The residue was purified by silica gel column chromatography (ethyl acetate /petroleum ether = 7:20) to afford compound **3** (3.26 g, 60%) as a white solid. ^{1}H NMR (400 MHz, $CDCl_3$) δ 4.72 (s, 1H), 3.89 (s, 2H), 3.45 – 3.37(m, 2H), 3.28 – 3.20 (m, 2H), 2.38 – 2.30 (m, 2H), 2.20– 2.10 (m, 2H), 2.03 – 1.84 (m, 2H).

To a stirred solution of compound **3** (2.71 g, 10 mmol) in dichloromethane (40 mL) was added S-(-)-Binaphthol (143 mg, 0.50 mmol), $Ti(OiPr)_4$ (71 mg, 0.25 mmol), H_2O (90 μL, 5.0 mmol) at 25°C. The mixture was stirred for 45 min, then *t*-BuOOH (70% in H_2O, 2.34 mL, 20 mmol) was added, and the reaction mixture was stirred for 4 h at 25°C. The reaction mixture was diluted with H_2O (40 mL) and ethyl acetate (100 mL). The layers were separated, and the aqueous layer was extracted with ethyl acetate (2×50 mL). The combined organic layers were washed with brine (2×20 mL), dried over Na_2SO_4, filtered and concentrated. The residue was purified by silica gel column chromatography (dichloromethane /methanol = 25:1) to afford the target compound **4** (2.3 g, 80%) as a white solid. ^{1}H NMR (400 MHz, DMSO-d_6) δ 8.67 (s, 1H), 3.70 (q, J = 11.1 Hz, 2H), 3.61 –3.51 (m, 1H), 3.36 – 3.28 (m, 1H), 3.18 – 3.08 (m, 1H), 3.07 – 2.98 (m, 1H), 2.32 – 2.15 (m, 4H), 1.83 – 1.69 (m, 2H).

To a solution of compound **4** (605 mg, 2.1 mmol) in tetrahydrofuran (20 mL) were added H_2O (5 mL), compound **5** (585 mg, 2.5 mmol) and *N*,*N*-Diisopropylethylamine (0.42 mL, 5.2 mmol) at room temperature, the resulting mixture was heated to 65 °C and stirred for 4 h. Then the reaction mixture was cooled to room temperature and concentrated under reduced pressure. The crude product was diluted with H_2O (50 mL) and dichloromethane (100 mL), the layers were separated, and the aqueous layer was extracted with dichloromethane (2×50 mL). The combined organic layer was washed with brine (2×30 mL), dried over Na_2SO_4, filtered and concentrated. The residue was purified by silica gel column chromatography (dichloromethane /methanol = 20:1) to afford BI 1015550 (707 mg, 75%) as a white solid. ^{1}H NMR (400 MHz, $CDCl_3$) δ 8.61 (s, 2H), 6.46 (s, 1H), 4.81 (br d, J = 8.6 Hz, 2H), 4.43 (t, J = 5.3 Hz, 1H), 3.78 (d, J = 5.3 Hz, 2H), 3.65 – 3.53 (m, 1H), 3.37 – 3.27 (m, 1H), 3.20 – 3.11 (m, 1H), 3.10 – 2.92 (m, 4H), 2.42 – 2.30 (m, 2H), 2.26 – 2.13 (m, 2H), 2.10 – 2.00 (m, 2H), 1.96 – 1.75 (m, 4H). ^{13}C NMR (101 MHz, $CDCl_3$) δ 174.81, 170.64, 162.46, 159.09, 155.65, 129.22, 107.58, 68.47, 59.20, 49.56, 44.97, 44.50, 32.86, 30.75, 30.26, 30.16, 14.96. HRMS (ESI) calculated for $[M+H]^+$ $C_{20}H_{26}ClN_6O_2S^+$ 449.1526, found 449.1521.

Figure S1. High Reslution Mass Spectrometry

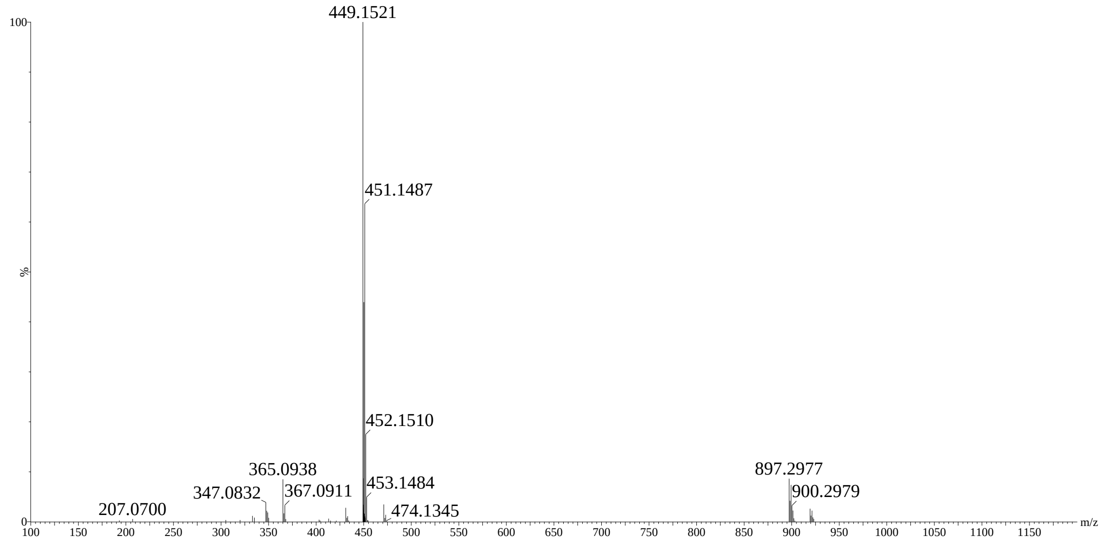

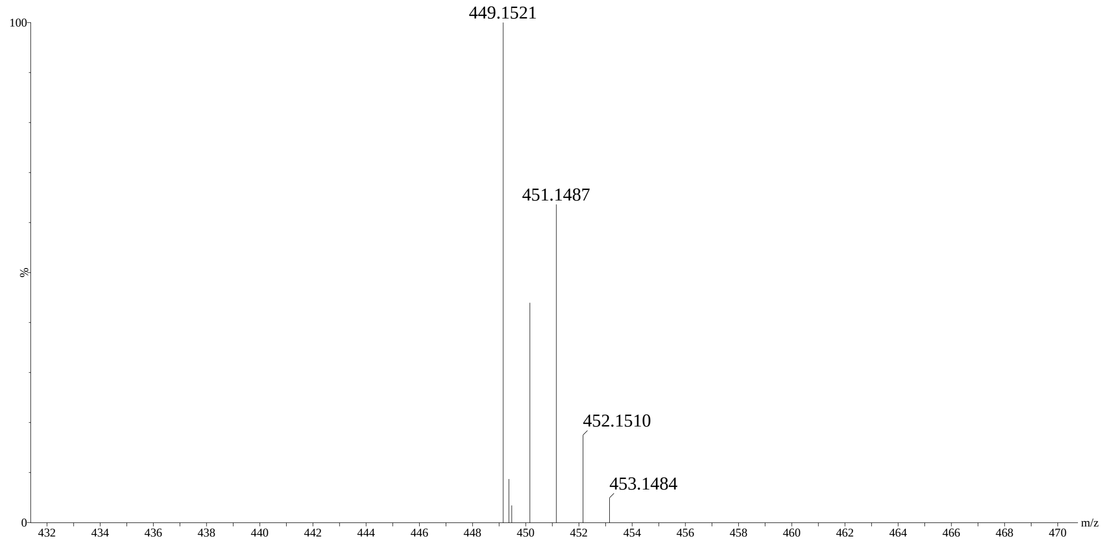

Figure S2. NMR Spectra

^{1}H NMR of **3** in $CDCl_3$

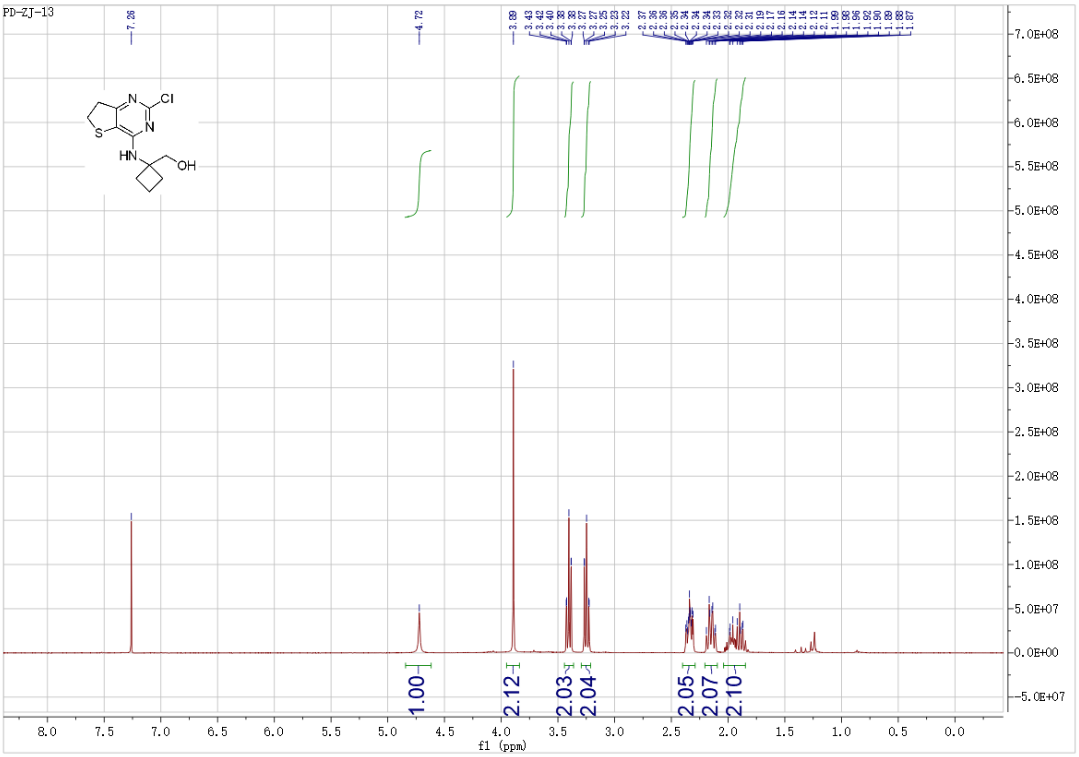

^{1}H NMR of **4** in DMSO-d_6

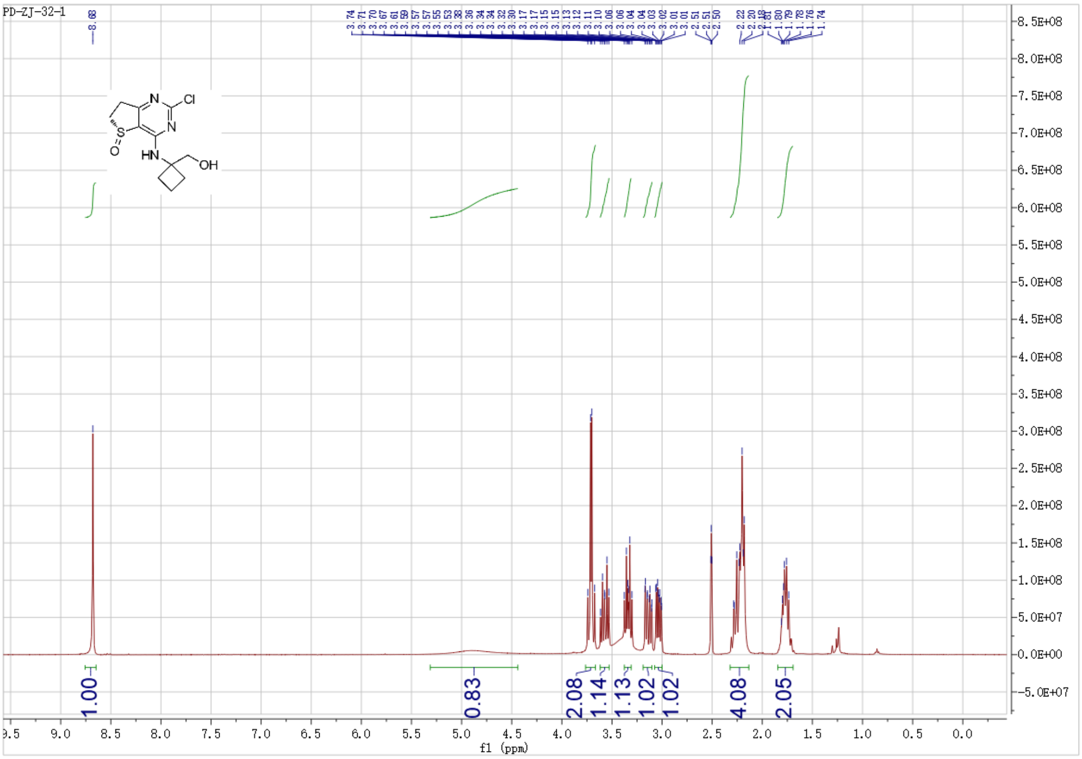

^{1}H NMR of **BI 1015550** in $CDCl_3$

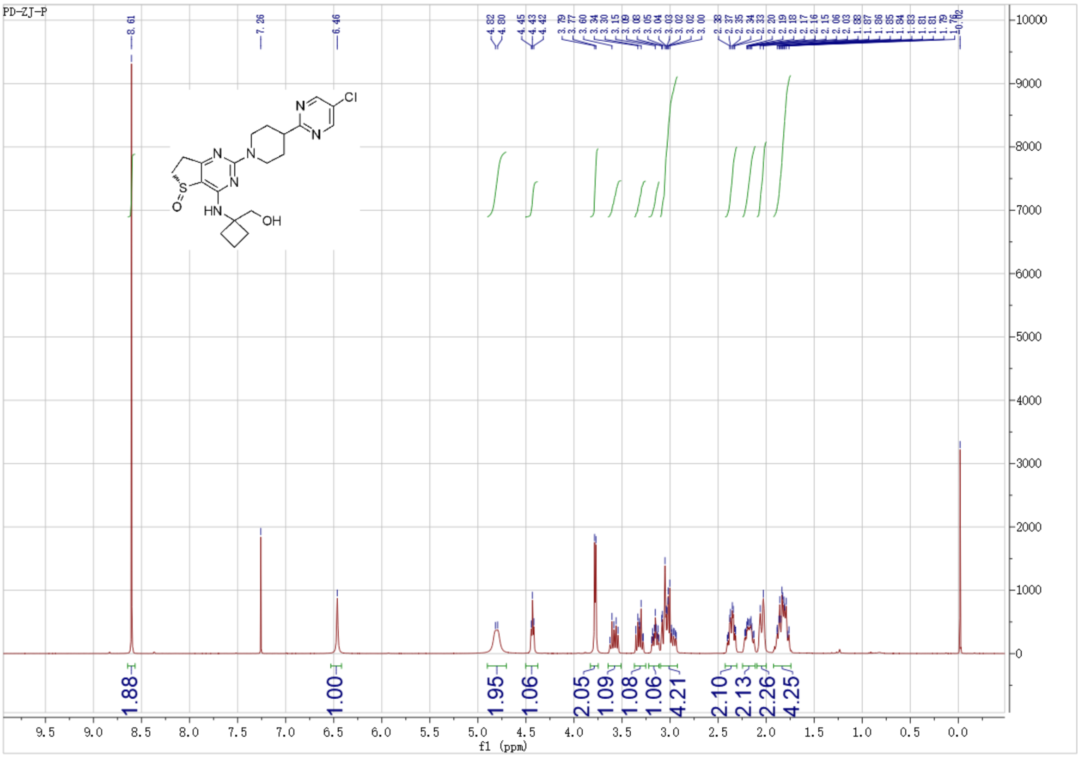

^{13}C NMR of **BI 1015550** in $CDCl_3$

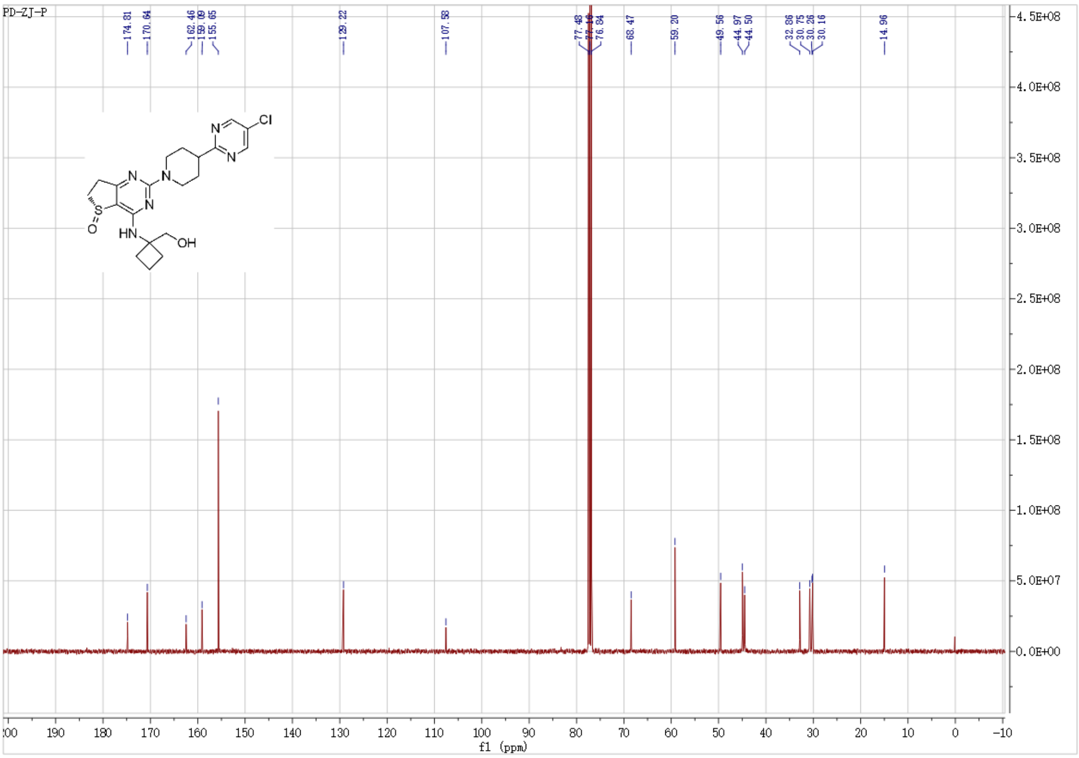
PD-ZJ-P
174.81
170.64
162.46
159.09
155.65
129.22
107.58
77.48
77.16
76.84
68.47
59.20
49.56
44.97
44.50
32.86
30.75
30.28
30.16
14.96
Cl
N
N
N
N
N
S
O
HN
OH
f1 (ppm)
